# Impact of infrasound exposure and streptozotocin-induced glucose intolerance on bone composition in Wistar rats

**DOI:** 10.1186/s13104-024-06784-x

**Published:** 2024-05-06

**Authors:** Luísa Zagalo, Gonçalo Pereira, Diogo Casal, Luísa L. Gonçalves, Carlos Zagalo, Maria João Oliveira, Pedro Oliveira, José A. A. Brito

**Affiliations:** 1 Center for Interdisciplinary Research Egas Moniz (CiiEM), Monte da Caparica, Portugal; 2 Plastic Surgery Consultant - Central, Lisbon University Hospital Centre, Lisbon, Portugal; 3 Department of Anatomy and UMIB - Unit for Multidisciplinary Research in Biomedicine, ICBAS - School of Medicine and Biomedical Sciences, University of Porto, Porto, Portugal; 4 ITR - Laboratory for Integrative and Translational Research in Population Health, Porto, Portugal; 5 Anatomy Institute, School of Medicine, University of Lisbon, Lisbon, Portugal; 6 CINTESIS@RISE, University of Porto, Porto, Portugal

**Keywords:** X-ray fluorescence spectroscopy, Elemental concentration, Infrasound, Streptozotocin-induced diabetes, Bone

## Abstract

The elemental composition of chemical elements can vary between healthy and diseased tissues, providing essential insights into metabolic processes in physiological and diseased states. This study aimed to evaluate the calcium (Ca) and phosphorus (P) levels in the bones of rats with/without streptozotocin-induced diabetes and/or exposure to infrasound. X-ray fluorescence spectroscopy was used to determine the concentrations of Ca and P in Wistar rat tibiae samples.

The results showed a significant decrease in bone P concentration in streptozotocin-induced diabetic rats compared to untreated animals. Similarly, the Ca/P ratio was higher in the streptozotocin-induced diabetic group. No significant differences were observed in bone Ca concentration between the studied groups or between animals exposed and not exposed to infrasound.

Moreover, streptozotocin-induced diabetic rats had lower bone P concentration but unaltered bone Ca concentration compared to untreated rats. Infrasound exposure did not impact bone Ca or P levels. The reduced bone P concentration may be associated with an increased risk of bone fractures in diabetes.

## Introduction

Phosphorus and calcium concentrations can vary between healthy and diseased individuals, affecting metabolic processes and pathophysiology in tissues such as bone [1–3]. However, studying individual elements in isolation can miss potential interactions between them [1]. Univariate methods are often used to compare healthy and diseased tissues or to correlate elements, but this can result in an inability to detect their effects [4, 5].

Alterations in the metabolism of glucose can be harmful to bone health, and both type 1 and type 2 diabetes mellitus have an increased risk of osteoporotic fracture as chronic complication [6, 7]. Bone plays a crucial role in regulating intermediary metabolism, making it a pathophysiological factor in the disease process itself [8, 9].

Infrasound exposure is increasing worldwide and this stimulus may affect bone remodeling and mineralization, possibly by stimulating the growth and secretion activity of osteoblast-like cells and promoting osteogenesis [10–15]. Little is known about the effects of diabetes and infrasound on elemental bone composition and their potential interaction.

Therefore, the aim of this study is to evaluate the elemental composition of Ca and P in the bone of streptozotocin-induced glucose-intolerant and/or infrasound-exposed rats using X-ray fluorescence spectroscopy [1, 2, 4, 5, 8, 16–18], using multivariate methods.

## Materials and methods

### Animal experiments

This study adheres to the 3Rs principles [19] and shares resources with a separate investigation of the effects of infrasound on pancreatic function and morphology, using the study design and sample size estimation previously described due to the expected effects [20]. Animal experiments were authorized by the Ethics Committee of Instituto Universitário Egas Moniz, the Portuguese National Authority for Animal Health (project nº 204/2017), and the Animal Welfare Body (ORBEA) of School of Medicine and Biomedical Sciences, ICBAS, University of Porto (Portugal), under protocol nº 204/2017. The experimental design complies with the PREPARE guidelines [21], and all animals were handled by FELASA Category C-accredited researchers and housed in a certified animal facility. The EU Commission on Animal Protection for Experimental and Scientific Purposes (2010/63/EU) and Portuguese legislation (DL 113/2013) were followed for animal care. This research followed the ARRIVE guidelines [22].

#### Animals

For this research, 133 male wild-type Wistar rats, aged 11 weeks, and weighing 375.95 g ± 18.29 g, were obtained from Charles River Laboratories (Saint-Germain-sur-l’Arbresle, France). All animals were male to avoid uncertainty in the results, due to sex-induced differences. The Preyer reflex test was used to evaluate auditory function in all animals [23], so that this factor would not act as a confounder. Rats were housed individually or in pairs in standard cages with unrestricted access to water and standard commercial rat chow under a 12-hour light/dark cycle. Housing conditions were maintained unchanged throughout the experiment. After a one-week acclimatization period, rats were randomly assigned to G1 (no treatment) or G2 (streptozotocin-induced diabetes) using open access online software [24].

#### Streptozotocin-induced diabetes

Initially, half of the animals randomly assigned were fed a high-fat diet (D12492 diet, Research Diets Inc., USA) for 3 weeks, comprising 60% fats, 20% carbohydrates and 20% protein (5.21 kcal/g energy density), compared to the standard rat chow (D10001 diet, Research Diets Inc., USA), which had 12% fats, 67% carbohydrates, and 21% protein (3.86 kcal/g energy density). Following the high-fat diet, rats were injected intraperitoneally with a low dose of streptozotocin (STZ, Sigma-Aldrich, USA) 40 mg/kg in a sodium citrate buffer 50 mM, pH 4.4, after fasting for 6–8 h, as described by Furman (2015) [25]. In this group of animals, glucose intolerance was confirmed using an intraperitoneal glucose tolerance test (glycemia ≥ 140 mg/dL at 2 h), following Ayala et al. [26]. Then, animals were randomly assigned to one of four groups: G1s (no treatment, silence), G1n (no treatment, infrasound), G2s (glucose intolerance, silence), and G2n (glucose intolerance, infrasound) groups. The animals from each of the four groups were sacrificed at 1, 6, or 12 weeks. The animals were chosen randomly for sacrifice at each timepoint.

#### Infrasound exposure

In the two groups exposed to infrasound, this exposure was implemented as previously described [27]. The enclosures were located in a soundproofed room with a noise generator consisting of a subwoofer, which produced a white noise signal filtered to generate high-intensity infrasound. The acoustic pressure waveform was examined, and the results showed an average sound pressure level of 120 dB in the 2–20 Hz range with a tolerance of ± 3 dB in a 30-second timeframe in the entire compartment. The groups not exposed to infrasound were held in a similar, quiet room.

#### Tissue and blood samples

To evaluate the evolution of glucose intolerance with infrasound factor compared to silence, glucose intolerance tests were performed after 1, 6, and 12 weeks of noise exposure using intraperitoneal administration, as described by Ayala et al. [26]. Glycemia time courses and area under the curve (AUC) were recorded, and plasma insulin levels were measured before and 30 min after glucose injection using a commercial ELISA kit [26]. Sacrifice by euthanasia was performed using carbon dioxide, and both hind limbs were manually removed for tissue sampling. Muscle, cartilage, periosteum, and tibiae samples were lyophilized for 48 h at -50 °C to minimize water content and optimize determination of elements of interest. Samples were then ground into an average particle size of 180 μm and kept cool, with masses ranging from 0.7149 to 1.1910 g.

### X-ray fluorescence spectroscopy

Tibiae bone samples were analysed using Wavelength Dispersive X-ray Fluorescence Spectrometry (WDXRF), a direct method with high sensitivity and detection limits [34]. Ca and P concentrations were determined using a 4 kW X-ray fluorescence spectrometer (S4 Pioneer, Bruker AXS) equipped with a Rh X-ray tube and a 34 mm diameter collimator mask. Polyethylene cups (35.8 mm diameter) with a 4 μm Prolene® film were used to hold the bone powder sample. Calibration was done with synthetic standards and bone matrix effects were simulated using a mixture of calcium carbonate and disodium hydrogen phosphate. Validation followed ICH guidelines [28] for specificity, linearity, detection and quantification limits, precision, and accuracy. Detection limits were estimated at 0.6% for Ca and 0.2% for P, and intra-assay precision was below 5% for both elements. Accuracy was established by measuring certified reference material (Caprine Bone NYS RM 05 − 01/ 05 − 04).

### Statistics

As elemental concentrations and ratios were determined in the same animals, a multivariate approach was initially considered due to potential intercorrelations between the variables. However, the Kaiser-Meyer-Olkin measure (0.257) indicated that this was unnecessary for our data. Therefore, univariate general linear models (GLM) were used to evaluate the effects of exposure and metabolic condition on bone composition, after selecting appropriate covariates. Normality and homoscedasticity assumptions were checked using the Shapiro-Wilk and Levene tests, respectively. The Statistical Package for Social Sciences (SPSS; IBM SPSS Statistics. Version 26.0, Armonk, NY: IBM Corp.) was employed for statistical analysis. The Pearson correlation coefficient was used to evaluate correlations between variables. Statistical significance was set at 5%.

## Results

To account for the effects of concomitant variables in the relationships between infrasound exposure and metabolic condition and bone composition, covariates were considered, including animal age, protocol duration, body weight, and AUC from OGTT. Insulin data were incomplete due to limited plasma samples, and AUC at baseline was considered as a covariate for Ca and P concentrations in bone. Normality and homogeneity of variances were violated for P concentrations, but GLM was still considered valid. The interaction between the covariate and main factors was non-significant. Table 1 presents descriptive statistics of bone composition variables for animals with complete records (*n* = 86) [29]. 

**Table 1:** Mean and SD of Ca, P and Ca/P in bone of animals with different status of glucose metabolism (G1/G2: no treatment/glucose intolerance) and infrasound exposure (Gs/Gn: silence/infrasound), with G1s (no treatment, silence), G1n (no treatment, infrasound), G2 (glucose intolerance): G2s (glucose intolerance, silence), and G2n (glucose intolerance, infrasound)

Group	Ca (%)		*P* (%)		Ca/*P*		
	Mean	Std. Deviation	Mean	Std. Deviation	Mean	Std. Deviation	N
G1s	23.44	1.80	7.61	0.76	3.09	0.25	24
G1n	23.90	1.51	7.63	0.76	3.15	0.28	22
G2s	22.87	1.51	7.02	0.83	3.29	0.29	24
G2n	22.62	1.54	7.15	0.79	3.19	0.29	16
G1 ( G1s + G1n)	23.66 ^a^	1.66	7.62 ^b^	0.75	3.12 ^c^	0.26	46
G2 ( G2s + G2n)	22.77 ^a^	1.51	7.07 ^b^	0.81	3.25 ^c^	0.29	40
Gs ( G1s + G2s)	23.15 ^d^	1.67	7.32 ^e^	0.84	3.19 ^f^	0.28	48
Gn ( G1n + G2n)	23.36 ^d^	1.63	7.43 ^e^	0.80	3.17 ^f^	0.28	38

Significance of Mean differences between G1 and G2 groups: (^a^) *p* = 0.331; (^b^) *p* = 0.040; (^c^) *p* = 0.066. Significance of Mean differences between Gs and Gn groups: (^d^) *p* = 0.708; (^e^) *p* = 0.671; (^f^) *p* = 0.787

**Fig. 1:**
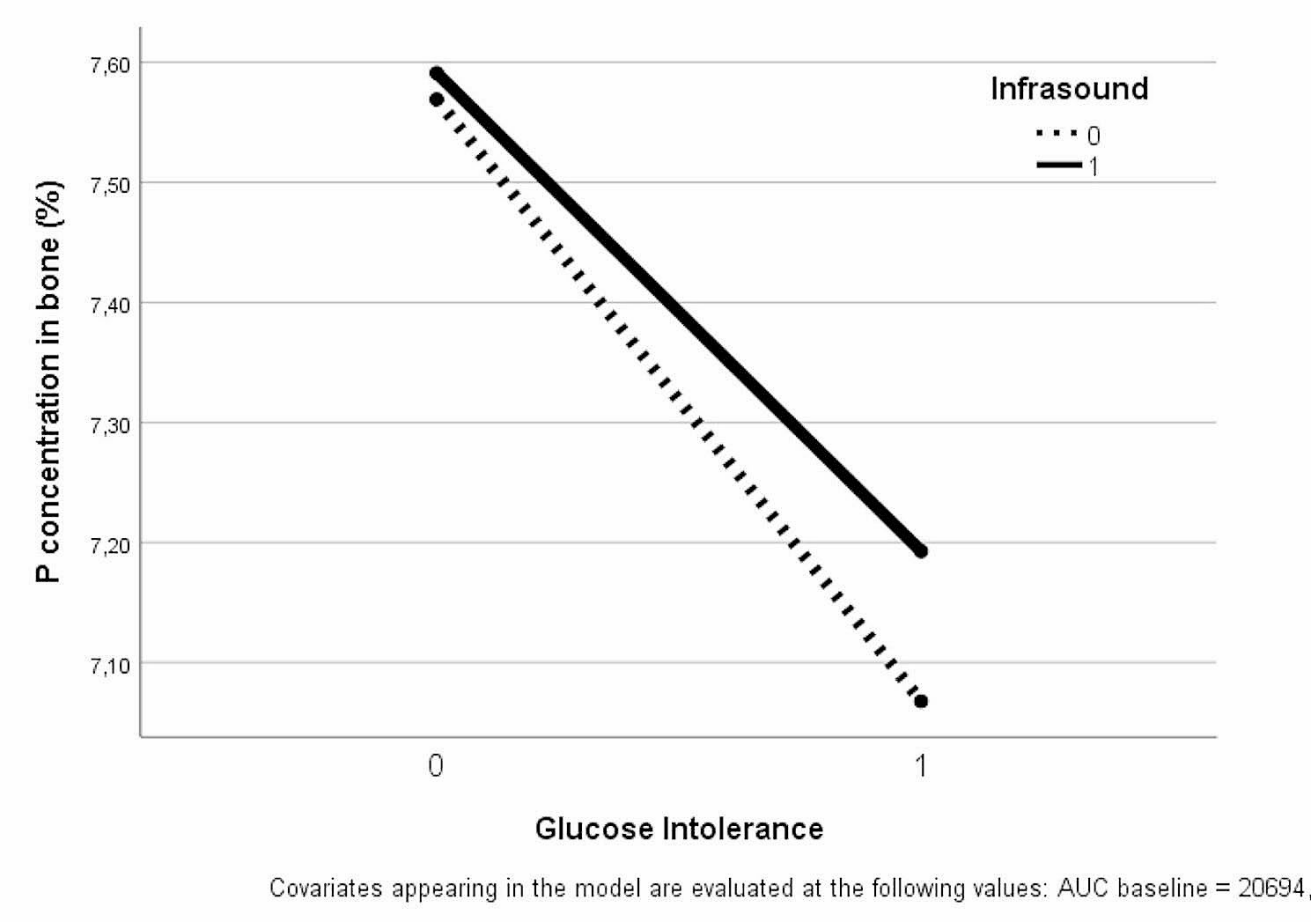
Profile plot of P concentration on bone of animals with different status of glucose metabolism and infrasound exposure, considered in GLM.

For Ca concentrations, the GLM approach found no interaction between main factors (*p* = 0.272) and no significant effects of exposure to infrasound (*p* = 0.708) or streptozotocin-induced diabetes (*p* = 0.331). Regarding P concentrations, the same approach found no interaction between main factors (*p* = 0.765) and no effect of exposure to infrasound (*p* = 0.671), but significantly lower P concentrations were observed in streptozotocin-induced glucose intolerant animals (*p* = 0.040) compared to non-glucose intolerant rats, regardless of exposure (Fig. 1). No significant effects were found for Ca/P ratios in bone due to interaction between main factors (*p* = 0.194) or to main factors, although higher ratios in the streptozotocin-induced glucose intolerance group were suggested but not statistically supported (*p* = 0.066).

Insulin, involved in controlling energy metabolism and bone mass [6], was considered in a subgroup of 52 animals with complete records including insulin at sacrifice, to assess its potential effects on the relationships between streptozotocin-induced glucose intolerance, infrasound exposure, and bone composition. Insulin was significantly associated with bone variables, showing an inverse correlation with P concentration (*p* = 0.003, *r* = -0.402) and a positive correlation with Ca/P (*p* < 0.001, *r* = 0.468). However, controlling for insulin does not change the significance of the effects of diabetes and exposure on bone concentrations and ratios observed in the larger subgroup.

## Discussion

Our study analysed Ca and P levels in rat bones exposed to streptozotocin treatment and/or infrasound. Findings indicate that glucose intolerance lowers P concentration and raises Ca/P ratio. There were no variations in bone Ca, P, or Ca/P ratio between infrasound-exposed and non-exposed rats.

Phosphorus deficiency in type 2 diabetes may suggest a glucose intolerance-induced phosphorus metabolism disorder, as serum phosphorus levels are known to decrease in these patients [30]. Our study found low bone phosphorus concentration, indicating that bones may release phosphorus to compensate for low serum levels, supporting the idea of altered phosphorus metabolism in glucose intolerance. Furthermore, hyperglycaemia significantly decreases parathyroid hormone levels [31], both of which independently contribute to reduced bone remodelling and quality [32].

Our investigation found no significant differences in bone calcium concentrations between the studied groups.

The low bone phosphorus and altered Ca/P ratio found in our study may be a predisposing factor for bone fragility in glucose intolerance and subsequently in diabetes. However, the importance of each variable to the clinical outcome situation is still uncertain [7].

Long-term exposure to industrial noise is known to disturb biological systems, and infrasound (< 20 Hz) is prevalent in the acoustic spectrum of industrial settings, but its effects on bone are still unknown [33]. Vibrations impact the function of osteoblasts and osteoclasts, reducing bone resorption and stimulating bone growth [12]. Exposure to vibration induced by infrasound may facilitate osteogenesis and fracture healing in vivo due to mechanical stimulation and increased activity of the bone neuro-osteogenic network [15]. The present study indicates that the streptozotocin-induced glucose intolerance did not affect the concentration of Ca, a major determinant of bone mass. In view of the spectrum of deleterious effects of diabetes on bone micro-architecture, the fact that bone Ca concentrations remained can speculatively be attributed to infrasound exposure, which somehow attenuated the bone effects of glucose intolerance. Therefore, we believe the infrasound exposure may have had a beneficial effect in the bone maintenance.

Changes in ion homeostasis in diabetic patients may be associated with increased morbidity and mortality [34].

A limitation of our study is the lack of full assessment methods of glucose homeostasis, such as insulin tolerance tests and islet function tests. These tests would offer a more complete picture of the metabolic status of the animals and further illustrate the deleterious effects of high-intensity infrasound exposure.

With this research we intend to investigate the effects of exposure to infrasonic noise and glucose intolerance on the bone composition of Wistar rats. Our results suggest that exposure to infrasonic noise may play a protective role in bone metabolism in the case of glucose intolerance. However, more studies are needed to clarify the effects of glucose intolerance and infrasound on bone metabolism.

## Conclusion

No differences in bone Ca were found between infrasound-exposed and unexposed animals or between glucose-intolerant non-intolerant animals. However, bone P concentrations were lower in glucose-intolerant animals with no difference between infrasound exposure groups. Glucose-intolerant animals had a higher Ca/P ratio than non-glucose-intolerant animals with no difference between infrasound exposure groups. This study is the first to describe elemental bone concentrations in glucose-intolerant animals exposed to infrasound, and the change in Ca/P ratio in glucose-intolerant animals. The observed low bone phosphorus may contribute to the bone fragility in glucose-intolerance or even diabetes, but further research is necessary to understand its mechanism.

## Data Availability

No datasets were generated or analysed during the current study.
